# Analytical Characterization and Sensory Analysis of Distillates of Different Varieties of Grapes Aged by an Accelerated Method

**DOI:** 10.3390/foods9030277

**Published:** 2020-03-03

**Authors:** Mónica Schwarz, M. Carmen Rodríguez-Dodero, M. Soledad Jurado, Belén Puertas, Carmelo G. Barroso, Dominico A. Guillén

**Affiliations:** 1 Instituto de Investigaciones Vitivinícolas y Agroalimentarias (Wine and Agrifood Research Institute) Analytical Chemistry Department, Faculty of Sciences, University of Cádiz, P.O. Box 40, Pol. Río San Pedro, 11510 Cadiz, Spain; maricarmen.dodero@uca.es (M.C.R.-D.); carmelo.garcia@uca.es (C.G.B.); dominico.guillen@uca.es (D.A.G.); 2 “Salus Infirmorum” Faculty of Nursing, University of Cadiz, 11001 Cadiz, Spain; 3 Instituto de Investigación y Formación Agraria y Pesquera (IFAPA) Centro Rancho de la Merced, Consejería de Agricultura, Ganadería, Pesca y Desarrollo Sostenible, Junta de Andalucía, 11471 Jerez de la Frontera, Spain; soljurado@hotmail.com (M.S.J.); belen.puertas@uca.es (B.P.)

**Keywords:** accelerated ageing, Brandy de Jerez, varieties of grapes, sensory analysis, distillate

## Abstract

The wine spirits used for the elaboration of Brandy de Jerez are mainly obtained from wines produced from the Airén type of grape, which comes from the vineyards located in the region of La Mancha (Central Spain). This entails a limitation when achieving a product classified as “protected geographic designation”. For that purpose, it is necessary that the grape used for the wine spirit comes from the area and not from Castile la Mancha, as has happened until now. Due to this fact, it is necessary to search for a possible alternative grape variety which allows the produced brandy to be eligible for a “protected geographic designation”. For that purpose, an accelerated ageing process has been implemented with a method previously optimized to distillates obtained from wines from different varieties of grapes (Airén, Colombard, Corredera, Doradilla, Garrido Fino, Jaén blanco, Moscatel de Alejandría, Palomino Fino, Ugni Blanc, and Zalema) grown in the Jerez Area. They were evaluated, both from the analytical and sensory points of view. The distillates made from Jaén Blanco and Zalema have properties that make them interesting for future development and incorporation into oenological practice.

## 1. Introduction

Brandy de Jerez is an aged distillate produced in the southern region of Spain according to the specifications included in the technical file of the protected geographic designation under such name [1]. Although it was and still is the spirit with the highest economic relevance in Spain, its marketing has dropped during the last decade 80%, from 45 million liters in 2008 to a bit over 9 million in 2018 [2]. This evolution may be due to several factors: the economic crisis during this period may have affected the figures too, but above all, this may be due to the increasing trend towards healthier habits, as the demand for wines, liquors, and spirits of a lesser alcoholic content has increased. In fact, many wineries of the Jerez area have replaced some of their marketed brandies by spirits with an alcoholic content between 30% and 33%. As they are not subject to the protected geographic designation, they are free to implement different practices to improve their profit margins, as the use of alcohol from a different source to wine or alternative ageing methods. 

Brandy de Jerez is aged in wooden casks following the traditional dynamic system (Soleras and Criaderas). This dynamic ageing involves the periodic racking of part of the contents of each barrel into another one containing older brandy, over the course of several years. The barrels are arranged in a series of scales, known as criaderas, ranked according to the age of the brandy contained. The final scale of the system is known as the solera. The fully aged brandy is drawn off periodically from it for bottling and sale. The wooden cask (American oak) serves both as a container during the ageing period and as an active contributor to the organoleptic properties of the product by means of the extraction of compounds from the wood. One of the characteristics differentiating Brandy de Jerez from others is that the casks in which it is aged have previously contained Sherry wine of one type or another: Fino, Oloroso, Pedro Ximenez, etc. This process is known as the “seasoning” of the casks and adds distinctive and unique organoleptic characteristics to Brandy de Jerez. The effect of this ageing in Brandy de Jerez has been well characterized in previous studies [3,4,5].

This ageing process is an expensive one, which leads to the search for alternative techniques which can speed up this ageing stage. The use of oak chips, assessed in different conditions (the amount of chips to be used [6,7], the type of used wood [8], the amount of added oxygen [9], or tools which may improve the extraction, such as an ultrasound [10]), have been studied to optimize different methods to age wines [6,7,8,9] or vinegar [11,12]. Thus, the ageing process has suffered some modifications lately, being the use of oak chips to accelerate the ageing process is a widely used practice in oenology.

There were not many references for distillates up to recent years. However, the number of studies evaluating accelerated ageing processes in distillates has significantly risen lately. There are studies optimizing accelerated ageing methods for plum distillates [13], sugar cane distillates [14], cider Brandy [15], or wine distillates [16], assessing analytical parameters which allow us to compare them with traditional cask ageing. For Brandy de Jerez, our research team has prepared and implemented a laboratory scale method using oak chips, previously soaked in Sherry wine to simulate the seasoning of the casks and an ultrasound to enhance extraction [17].

The ageing of distillates is a stage where many transformations take place. Among these transformations, we can point out the evolution in polyphenolic content and color [18,19], together with significant modifications in the flavor of the aged product [16,20].

The physical and chemical processes which lead to these changes (wood extraction, oxidation enhanced by atmospheric oxygen, esterification, hydrolysis, and ethanolysis) depend on the composition of the wood and on the atmospheric conditions but also on the young distillate, as, together with presenting its own characteristic aromatic profile, will condition the interactions with the wood and the oxygen depending on other variables of its composition, like pH, acidity, or alcoholic content. As a result, the type of grape used to prepare the distillate affects the quality of the final product. Thus, although most of the research studying in-depth the ageing process of the distillates focuses on assessing the used wood or the ageing time, there are some studies which have proven the influence of the used grape variety [21,22]. 

The varieties used to obtain the distillates which will be later on be aged to obtain Brandy de Jerez are Palomino Fino and Airén grapes, being the latter the most common one. This type of grape (Airén) is grown mainly in Castile La Mancha, a region located 600 kilometers away from the Jerez area. The reason for the use of wines of this type of grape is mainly due to the fact that it is the most widely grown type in Spain (around 500,000 ha), and in a way, a use for the wine surplus produced in that area was found. 

The use of Airén as the most common grape variety, for the elaboration of Brandy de Jerez sets a limitation when achieving a product certified as belonging to a protected geographic designation (DOP, according to its initials in Spanish). This is a label used to legally protect certain food or foodstuffs produced entirely in a specific area against producers from other areas wanting to take advantage of the good name created thanks to a long manufacturing or breeding time of the original ones. To do so, the designation demands that the used grape belongs to the production area and not to another one (Castile La Mancha), as has happened up to now. 

On the other hand, it is widely accepted that the product diversification strategy can be useful in order to improve the competitiveness of the companies as they meet the demands of different types of consumers. Would a Brandy de Jerez with higher fruity and floral notes, produced from a more aromatic grape variety, be accepted by the public?

Due to this, it is important to search for an alternative to the aromatically neutral Airén grape, which can on one side improve the quality of brandy, increasing its personality, and on the other, allow Brandy de Jerez to apply for a protected geographic designation, if this grape variety were a native species of such area. 

The aim of this study is, therefore, to assess from an analytical and sensory point of view the way in which distillates made from different grape varieties age. Thus, it will be possible to identify those which render better results and which could be useful in future research to pose an alternative to the Airén grape type. In order to do so, distillates from wines obtained from different grape types (Airén, Colombard, Corredera, Moscatel de Alejandria, Ugni blanc, and the highly productive and native from Andalusia Doradilla, Garrido Fino, Jaén Blanco, and Zalema, besides Palomino Fino, also from the area) grown in the Jerez area have been aged through the accelerated ageing method prepared in previous studies [17].

To do so, two specific objectives are presented:

1. Comparing the physical-chemical profiles of the varietal distillates aged in an accelerated way during one month with those for a Brandy de Jerez aged in the traditional Solera and Criaderas system, which will serve as a model to assess the ability of each varietal distillate to reach a given physical-chemical profile associated to age. The assessment of the speed with which this process takes place is also highly interesting, as it will be a relative time estimate for each varietal distillate in a traditional, variable aging, significantly affecting the cost of the process. Additionally, this would allow us to have the required time data to age the varietal distillates in an accelerated way to be marketed under the spirits branding category within the scope of the product diversification strategy by the wineries.
2. Analyzing the aromatic sensory profiles of the varietal distillates after one month of accelerated ageing, comparing them to young distillates before the ageing, so as to assess their evolution and final quality.

## 2. Materials and Methods

### 2.1. Samples

A total of 10 wine distillates made from just one grape variety each from the same harvest and produced by Rancho de la Merced (IFAPA center from Jerez de la Frontera) were subjected to an accelerated ageing process through a previously developed method [17]. The used types were: Airén, Colombard, Corredera, Doradilla, Garrido fino, Jaén Blanco, Moscatel de Alejandria, Palomino Fino, Ugni blanc, and Zalema, all of them grown in such research center [23]. The ageing distillates were compared to a series of samples of brandies from a pilot experience carried out by our group in the past in collaboration with the specific regulatory council of Brandy de Jerez [5,24]. The reference samples were divided into three groups depending on their average ageing time, i.e., Solera (S, aged in oak casks for a minimum of 6 months); Solera Reserva (SR, aged for a minimum of 1 year); and Solera Gran Reserva (SGR, aged for a minimum of 3 years). In total, 144 samples were analyzed (80 accelerated ageing samples and 64 traditional ageing system samples).

### 2.2. Accelerated Ageing Process

The samples were aged by means of the method prepared in previous researches [17]. The amount of wood chips used in this method was double the surface/volume ratio of a Sherry wineskin (64 cm^2^/L). To optimize this amount, 100 chips were measured and weighted, taking an average of them. Then, the chips were wined in Oloroso Sherry wine, so as to imitate the traditional seasoning of the wineskins for Brandy de Jerez. The accelerated ageing was carried out in 150 mL test tubes with wine spirit and oak wood chips, labeled and packed in a tea-like bag. These bags were immersed into the probe and subjected to stirring and ultrasound for 30 days continuously. Stirring took place by means of OVAV magnetic stirrers (Barcelona, Spain), and ultrasound was performed by means of JPSELECTA with a water recirculating device for the control of temperature (20 °C) (JULABO F12) (Barcelona, Spain). Twice per week, samples were taken from the accelerated ageing method. In each sampling, 2 mL of wine spirit were taken, replacing them with 2 mL of the initial wine spirit. This method [17] optimized the sampling with two main aims: to control the assessment of the ageing process of the distillate and, on the other hand, to simulate the dynamic ageing process of distillates in Jerez, explained in Section 1. Oak chips were supplied by Nutritec (Barcelona, Spain). For further details on the accelerated ageing process, please refer to previous articles [17].

### 2.3. Analysis of Polyphenols and Furanic Compounds

These analyses were performed on a Waters Acquity UPLC system coupled with a photodiode array detection method [25]. An Acquity UPLC BEH C_18_ column (100 × 2.1 mm/ID, with 1.7 μm particle size), also from Waters, was used. The column temperature was maintained at 47 °C. Acetonitrile (ACN) (Panreac, Barcelona, Spain) and acetic acid (Scharlau, Barcelona, Spain) used were of HPLC grade. Water was supplied by a Milli-Q water purifier system from Millipore (Bedford, MA, USA). The binary system phases were: A (3% ACN, 2% acetic acid, 95% water) and B (85% ACN, 2% acetic acid, 13% water), with a flow rate of 0.7 μL/min, giving a maximum back pressure of 10 400 psi, which is within the capabilities of the UPLC. The injection volume was 2.5 μL. The 6.5 min gradient was as follows: 0 min, 100% A, 3 min, 90% A, 4 min, 90% A, 6.5 min, 25% A. Finally, the column was washed with 100% B for 3 min and equilibrated with 100% A for 3 min. All the samples were filtered through 0.22 μm nylon filters from Scharlab (Barcelona, Spain).

Furaldehyde, hydroxymethylfuraldehyde, syringic acid, coniferaldehyde, and sinapaldehyde were from Sigma-Aldrich (Steinheim, Germany). Gallic and *p*-coumaric acids were from Merck (Darmstadt, Germany). Vanillin, syringaldehyde, and vanillic acid were purchased from Fluka (Buchs, Switzerland). The working standard solutions for linear calibration were dissolved in a brandy-like model solution, prepared with ethanol (Panreac) (40% *v*/*v*) and purified water. Each level of concentration for the calibration curve was performed in triplicate. The identification of each compound was carried out by comparing retention times and UV–Vis spectra of the peaks with those previously obtained by the injection of standards.

### 2.4. Color Measurements

The chromatic characteristics were determined according to the CIELab method following the Commission Internationale de L’Eclariage’s recommendations [26]. They were determined with a Helios UV-Vis spectrophotometer by measuring the transmittance every 10 nm from 380 to 770 nm, using a D65 illuminant and a 10° standard observer. All measurements were carried out in duplicate.

### 2.5. Analysis of Total Phenols

The total polyphenolic index was measured following the Folin–Ciocalteau method [27]. The reaction mixture contained 250 μL of sample, 1250 μL of Folin–Ciocalteau reagent (Sigma-Aldrich), and 5 mL of 20% sodium carbonate (Panreac). Dilutions were carried out in duplicate, and the absorbance was measured at 750 nm. The calibration curve was prepared with gallic acid solutions ranging from 0 to 1000 mg L^−1^, and the results are given as gallic acid equivalents (GAE).

### 2.6. Sensory Analysis

The sessions took place in a normalized test room for sensory analysis [28], where the influence of external factors on the obtained results was minimized. The temperature of the room was set to 22 °C. Assessments were solely carried out through orthonasal perception. The samples were not tasted in any case.

The panel was formed by 10 assessors (4 men and 6 women) whose ages ranged from 23 to 52 years old. All of them belonged to the staff of the Analytical Chemistry Department of the University of Cadiz and had previous experience in the sensory analysis of wines and similar products. Additionally, they all had a training period in general and specific aspects on the sensory analysis of distillates. 

A total of 5 training sessions were held. A preliminary study was implemented by means of a questionnaire on those aspects which might affect somehow their work as assessors (tobacco use and distillate drinking habits, timeliness, etc.), as well as physiological aptitude tests [29] and characterizations of the olfactory sensibility of the assessors by means of an ordering test [30], according to the directions of the standard [31]. The aged brandies were presented to the assessors in 3 sessions before their assessment, during the characteristic descriptor stage [32,33], which allowed them to become familiar with the samples. Aged distillates (7 samples) were presented to the assessors: 3 Brandy de Jerez samples of different ages (Airén type and aged in a traditional way) and 4 varietal distillates aged in an accelerated way (2 of them from low aroma grapes, Airén and Palomino Fino, and 2 made from more aromatic grapes, Zalema and Moscatel de Alejandría). The assessors were asked to describe the samples from a qualitative point of view. With these data, the descriptors appearing more than 5 times were selected for the study. In this way, the finally selected descriptors included in the tasting note were (Table 1): aromatic intensity, fruity, herbaceous, vinous, alcoholic, milk, sweet, wood, vanilla, toast, spices, chemical, humidity, animal, and olfactory perception (othonasally). As olfactory patterns of the selected descriptors, the assessors were offered some samples (belonging to this research or not) where the corresponding descriptor was perceived with a high intensity (5–6 points in a 6-point scale).

Once the panel had been trained, the descriptors had been generated, and the tasting note had been designed, the aged brandies belonging to this research were descriptively assessed. 

The duplicates of the descriptive tests of the study were used to validate the reproducibility of each assessor, whereas, for each descriptor, the ANOVAs of two factors (assessors × samples or assessors × weeks) allowed us to study the homogeneity of the panel.

### 2.7. Data Treatment

The analysis of variance (ANOVA) and the principal component analysis (PCA) were carried out by using the statistical computer package Statistica 7.0 (Tulsa, OK, USA). 

## 3. Results and Discussion

### 3.1. Analytical Characterization of the Distillates Aged in an Accelerated Way

#### 3.1.1. Color and Index of Total Polyphenols

**Figure 1:**
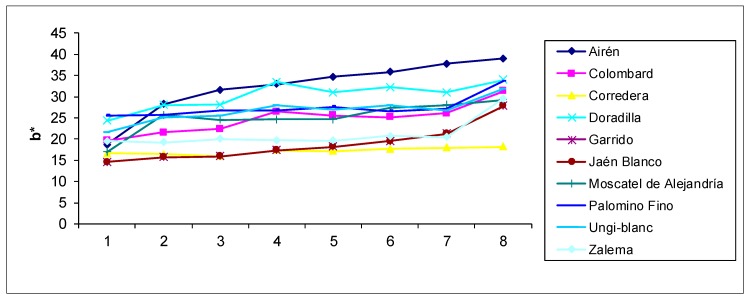
Evolution of the b* values in the aged distillates of the different varieties. Sample 1 corresponds to the first sampling, and sample 8 corresponds to the last one. b*: positive yellow-green tonality values.

**Figure 2:**
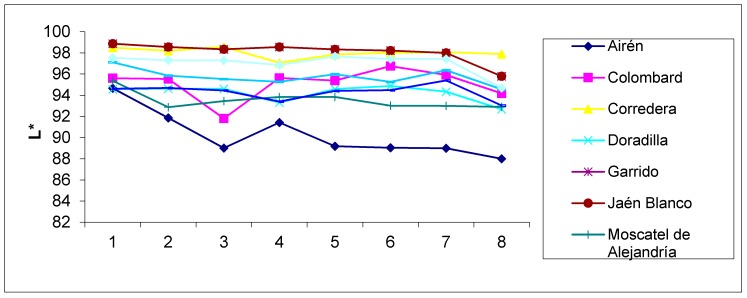
Evolution of the L* (brightness of color) values in the aged distillates of the different varieties. Sample 1 corresponds to the first sampling, and sample 8 corresponds to the last one.

In accordance with the values of the chromatic parameters, the samples aged for longer periods of time present a darker color (lower values of the L* brightness and H* tone parameters), with a more marked yellow–green tonality (negative a* values and positive b* values). Figure 1 and Figure 2 show the evolution of the b* and L* values, respectively, in the aged distillates of the different varieties. Sample 1 corresponds to the first sampling, and sample 8 corresponds to the last one. The figures show how parameter b* increases along the successive samplings, whereas luminosity drops in general. In the applied ANOVA, the differences according to the sampling time were significant for all chromatic parameters (*p* < 0.05) (Table 2). This evolution of chromatic parameters has also been found in other articles [17,34]. The a* chromatic parameter has negative values, increasing its absolute value with the sampling or, in other words, with the ageing time. This agrees with the sensory analysis of brandy in practice, which searches for greenish hues and links them to a longer ageing of the distillate. These negative values contrast with the positive ones of commercial Brandies de Jerez analyzed in previous cases [4]. This is due to different reasons, as ageing time and the type of wine stored previously in the barrels before ageing the Brandy de Jerez, or allowed practices, as the addition of caramel. 

As regards the chromatic differences according to each specific grape variety (Table 2), the ANOVA analysis confirms that there are differences regardless of the parameter (*p* < 0.05). The Airén distillate had a lower greenish hue than the rest, among which, no difference was detected, with a more present yellow hue, besides showing the lowest luminosity. Together with the Jaen Blanco distillate, which comes second, they are the samples with the highest intensity as regards color.

**Figure 3:**
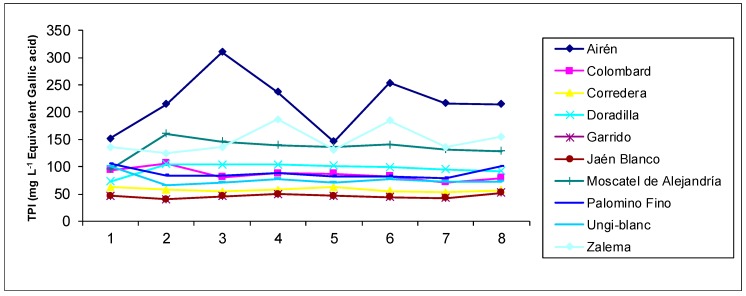
Evolution of the total polyphenol index (TPI) values in the aged distillates of the different varieties. Sample 1 corresponds to the first sampling, and sample 8 corresponds to the last one.

As regards the total polyphenol index (Figure 3), we can see how, in general, all varieties reach higher values in the first week and a half of the accelerated ageing process (the first three samples), only to decrease after that again. This meets the analytical characterization carried out to Brandy de Jerez which has been traditionally aged [4] and to accelerated aged grape distillates [17]. These differences as regards sampling were finally not significant from a statistical point of view (*p* = 0.126) (Table 2). However, differences were indeed significant among the different varietal distillates, being the distillate produced with Airén the one which showed the highest values, followed by the Jaen Blanco, in correlation with the lowest luminosity presented by these same samples.

All three figures reflecting the evolution of the chromatic parameters (Figure 1 and Figure 2) and the total polyphenol index (Figure 3) clearly show that the behavior of the aged distillates is different according to the used grape varieties, despite the fact that all of them have been aged in the same way.

#### 3.1.2. Polyphenols and Furanic Compounds

Furanic compounds and the polyphenols identified in the aged distillates coincide with those found in previous studies [4,15,19,34]. Their origin is different, though, being most of them related to the wood extraction [35]. Furanic aldehydes are also present in the original non-aged distillate due to the high temperatures reached during the distillation process, and the ANOVA data (Table 3) show that this source ended up being more significant than the extraction in our samples, as no difference according to the sample was proven (*p* > 0.05). 

Table 4 shows the concentrations of the distillates at the end of the accelerated ageing process. As can be seen, gallic acid, syringaldehyde, coniferylaldehyde, and sinapaldehyde are the most significant polyphenolic compounds. These results coincide with previous works by our research group [17], as well as with other authors who have aged different distillates [15,19]. These results show again significant differences among the behavior of the different varieties as compared to any of the furanic or polyphenolic compounds (*p* < 0.05). 

### 3.2. Comparison of Those Distillates Aged in an Accelerated Way with Traditionally Aged Brandy de Jerez Samples

**Figure 4:**
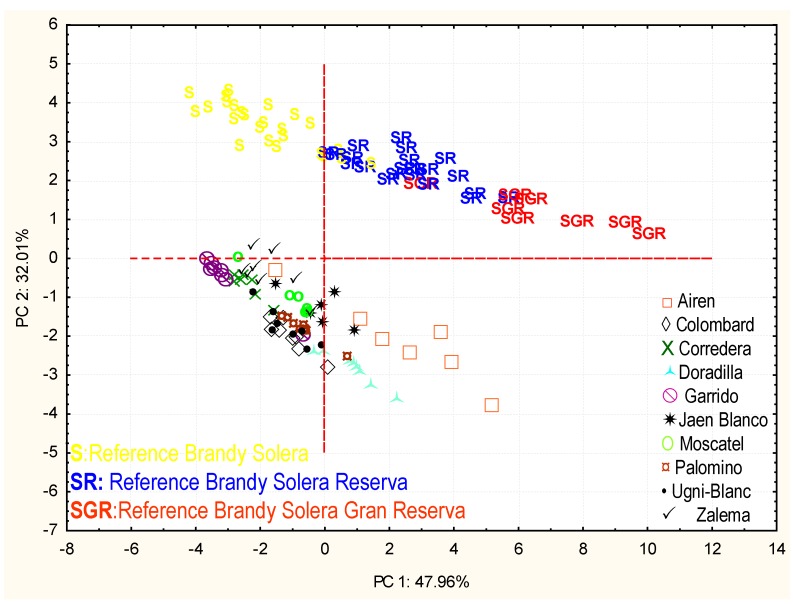
Principal component analysis with distillates of different varieties of grapes aged by an accelerated and traditional method. Projection of the cases on the factor plane.

**Figure 5:**
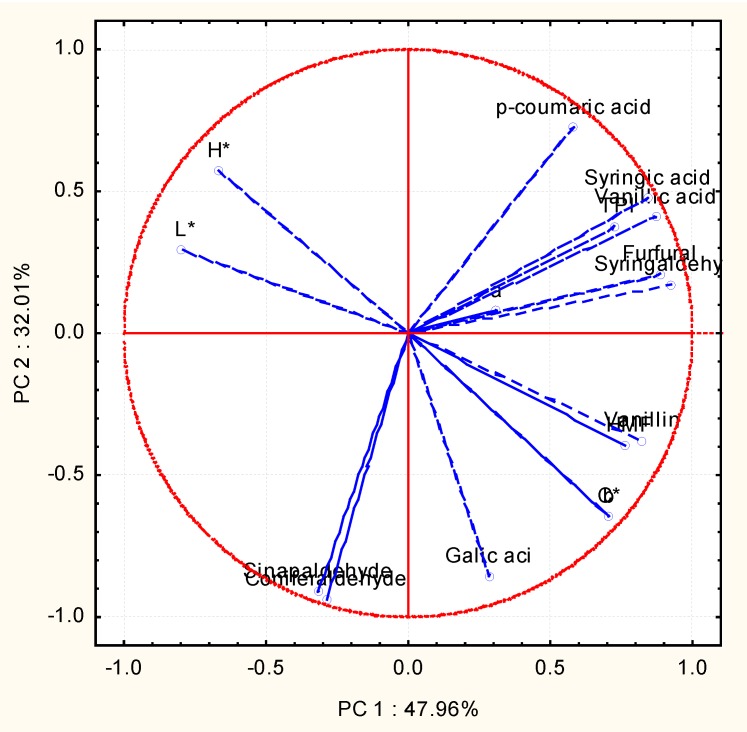
Principal component analysis. Projection of the variables on the factor plane.

A principal component analysis with all studied parameters was carried out on the set of samples: the distillates from the different aged types plus the reference samples from the Solera and Criadera system. The first two components explain 80% of the variability among the samples. Figure 4 shows how component 2 behaves differently in the samples which were aged in a traditional way and in those aged in an accelerated way. If the weight of the variables in these two first components is analyzed, we can see how the variables with the highest weight in principal component 2 are those polyphenols directly able to be extracted from wood: gallic acid, coniferaldehyde, and sinapaldehyde (Figure 5).

**Figure 6:**
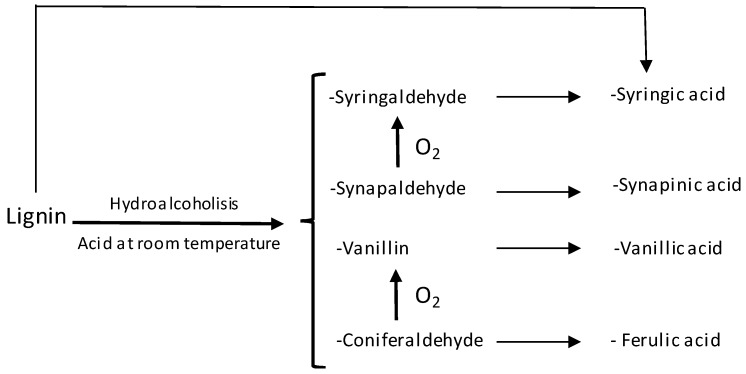
Formation and evolution of phenolic compounds from oak wood.

Previous experiences [17] make us think that this may be due to a high extraction of these elements by the wood chips in the accelerated ageing method, which might justify the differences between the samples which had been aged in a traditional way and those aged through the accelerated method along component 2. After analyzing the reaction mechanisms in the creation and evolution of the phenolic compounds coming from the oak wood (Figure 6) [35], we thought that the extraction of these compounds would be lessened if chips had been combined with oxygenation during the accelerated ageing process, as has been proven in other studies [36]. In this case, it could not be done due to the fact that the accelerated ageing method took place on a laboratory scale with a very scarce sample volume, which prevented us from being able to apply small volumes of oxygen. Besides, a larger extraction of polyphenolic compounds in accelerated ageing methods for distillates as compared to traditional methods has been also found in previous articles [34].

However, despite this difference, it is worth mentioning that the samples are ordered along component 1 according to their average age, being those to the left the least aged ones (solera) and the most aged ones, to the right. As a result, and according to the order in component 1, we can say regarding the aged distillates that the Airén type is the one which has achieved a most similar aging level to the Solera Reserva and Solera Gran Reserva samples, followed by the Jaén Blanco and Doradilla types, although the latter is located further down. 

**Figure 7:**
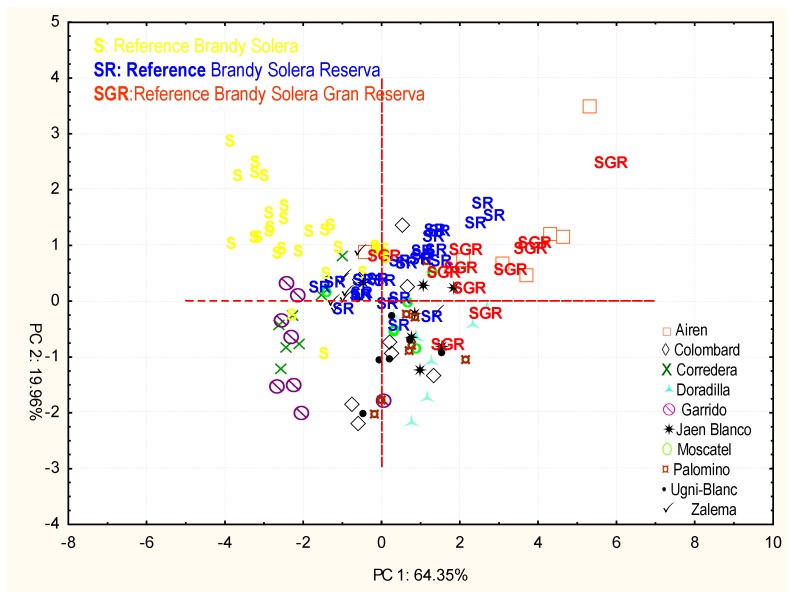
Principal component analysis with distillates of different varieties of grapes aged by an accelerated method and traditional method. Projection of the cases on the factor plane.

So as to prevent an excessive influence of the quick extraction compounds when devising the model, a principal component analysis was implemented on the samples, taking into consideration just the chromatic parameters and the total polyphenol index (Figure 7 and Table 5).

With the first two components, 84.31% of the variability among the samples was explained. As shown by the figure, the Airén grape type is located by the Solera Gran Reserva reference samples which had been aged in the traditional way in just a month of accelerated ageing. The aged samples from grape types Jaén Blanco, Moscatel de Alejandría, and Zalema are located next to the Brandy Solera Reserva reference samples which had been aged in the traditional way, whereas the rest of types are located on the left-bottom area of the graph, far from the behavior of the reference samples. 

### 3.3. Sensory Analysis

The young distillates and those which had been subjected to an accelerated ageing were analyzed after 30 days from a sensorial point of view, carrying out a descriptive assessment of each of them in duplicate. These duplicates were used to assess the repeatability of each of the assessors, so that a difference of no more than one point among the replicas of one sample were admitted. Besides, the homogeneity of the panel was confirmed with the results of the variance analysis of two factors applied to the samples and assessors, whose *p* values are included in Table 6. As can be seen, for the assessor factor, no values under 0.05 were reached for any of the descriptors, and taking into account that no significant interaction (*p*_assesorsxsamples_ > 0.05) was found, the similarity in the opinions of the assessors of the panel was confirmed. 

As can be seen in Table 6, the best perceived descriptors were aromatic intensity (with medium-high values from 3.8 to 5.4 on a 6-point scale) and notes alcoholic (2.9 to 4.2), fruity (2.0 to 4.6), and oak (0.4 to 3.3), reaching average values on the scale. Olfactory impression presented means within a 3.0 and 5.2 range (also on a 6-point scale), and the young distillates made of Ugni Blanc and Moscatel, respectively, lie in the outskirts of this scale. These results confirm those obtained by Jurado [23]. 

For each descriptor, two-way ANOVAs (varieties × ageing) have been implemented to find out which are the attributes best distinguishing the distillates of different varieties of grapes and whether these attributes are the same in the young distillate sample and in the aged one. As shown by Table 6, the *p*-values associated to the differences of the descriptors according to the varieties (*p_Varieties_*) are all over 0.05; i.e., from a statistical point of view, none of the descriptors makes a significant differentiation of the samples made from different grape types. Just the fruity note was close to showing a significant difference (*p_Varieties_* = 0.078), being the Moscatel distillates the ones showing a higher intensity, whereas the non-aged Ugni Blanc sample presented a much lower intensity to the rest of young distillates. 

When the ageing effect is analyzed, some descriptors significantly modify their values, and in a significant way, those with *p_agein_* under 0.05, in italics in Table 6. According to these results, the distillates increase their intensity regarding sweet, oak, vanilla, and toasted notes after ageing. As it is well known, these notes correspond with typical aromas of aged distillates in oak wood. On the contrary, the primary fruity aroma, related to the raw material, and the secondary aromas deriving from fermentation, vinous, and alcoholic, decrease. Regarding the herbaceous note, despite the apparent significance of the ageing factor (*p_ageing_* = 0.026), the results are not clear, as the interaction of the factors for this descriptor was significant (*p_ageing_* = 0.047), with a reduction of the average intensity with ageing for almost all distillates except for the Airén, Jaen Blanco, and Ugni Blanc distillates, where the average intensity increased. 

As regards the negative variables, the chemical note decreases and the animal one increases as the samples age (*p*-values under 0.05, in italics in Table 6). Nevertheless, the values close to the 0.05 threshold in the *p*-statistic for the interaction of the considered factors (*p_VarietiesxAgeing_*) mean that the trend is close to not being the same for all varietal distillates. In particular, and despite what has been mentioned before, the mean data indicate that the intensity of the chemical flaw increases very slightly with ageing in the Moscatel and Palomino Fino distillates, whereas the animal flaw decreases in the Ugni Blanc distillate. 

The theory of the authors is that the reason for these interactions is related to the low intensities characterizing these two descriptors (below 1 in most cases), together with the typical dispersion of the sensory assessment panel. Due to this, it would be necessary to implement further future research to confirm the presence of these defects in the distillates and whether their evolution through ageing depends on the type or grape or not. 

The olfactory impression does not show any significant differences, which makes it difficult to obtain any conclusions regarding it.

In view of the results, it could be a good choice to treat the data from a multivariate point of view. An exploratory (non-supervised) factorial analysis was implemented on the set of the young samples and the accelerated ageing ones. The first two components explain 67% of the variability among the samples. 

**Figure 8:**
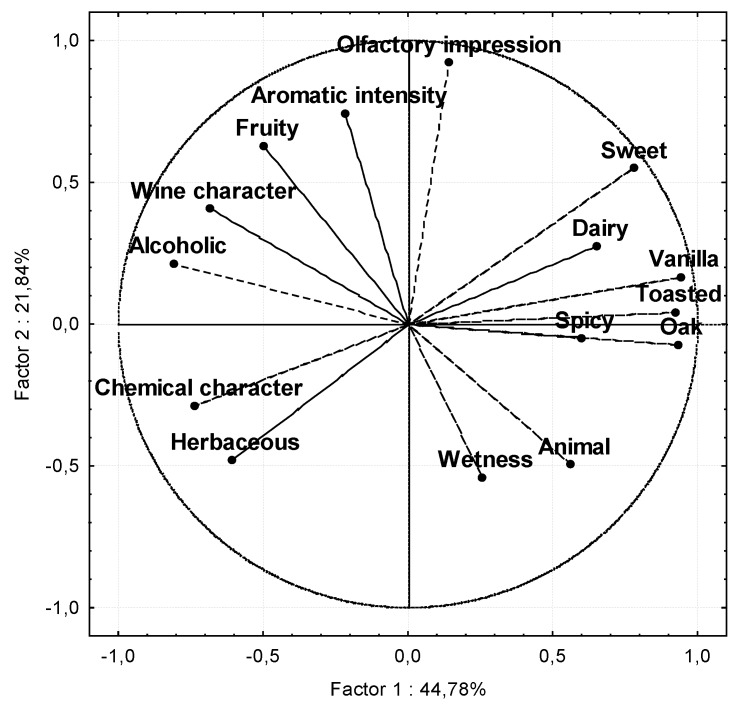
Principal component analysis of sensory data. Projection of the variables on the factor plane.

The first factor (Factor 1 in Figure 8) explains 44.78% of the original variance. Considering those normalized loads over 0.7 in absolute value, that is to say, values over ±0.7 on the axis x, it can be said that Factor 1 is directly correlated with the oak, vanilla, toasted, and sweet descriptors, and on the other hand (due to the negative sign), with alcoholic, chemical, vinous, and herbaceous. Meanwhile, the second factor (Factor 2 in Figure 8) explains 21.84% of the variance and presents a high positive correlation (with loads over +0.7) with the olfactory impression, aromatic intensity, and fruity note.

**Figure 9:**
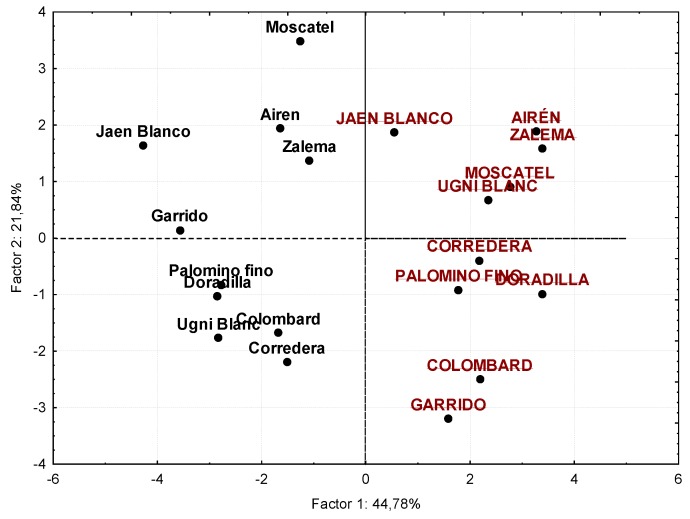
Principal component analysis of sensory data. Projection of the cases on the factor plane. Capital letters correspond to aged distillates and lowercase letters distillate to non-aged distillates.

The distribution of the samples on the new graph is shown in Figure 9, where a clear difference between the young (in black lowercase letters) and the aged (in red capital letters) samples can be seen. This distribution (to the left and right of Factor 1, respectively) clearly responds to the high weights of the oak, vanilla, and toasted descriptors in the positive values of Factor 1, as they are all aromatic notes related to the oak wood, provided to the distillates by the chips after being in contact with them. On the contrary, the primary and secondary descriptors herbaceous, vinous, and alcoholic, originating from the raw materials and fermentation, are placed on the negative x-axis, where young distillates are located.

On the other side, Factor 2 seems to be related to the quality of the aromatic profile, due to the significant positive contribution of descriptors such as aromatic intensity and fruity (both of them contributing to the complexity of the aroma) together with the olfactory impression. Thus, the situation of the distillates regarding Factor 2 allows the identification of the best-assessed varietal distillates and those with the highest aromatic intensity and fruity notes, which will be the ones with the highest values in Factor 2, i.e., those located in the highest area of axis y. According to this criterion, in Figure 9, the following distillates stand out: Moscatel de Alejandría, Airén, Jaen Blanco, and Zalema, both regarding young samples and aged ones. These results fully coincide with those obtained by Jurado [23] in her chemical and sensory study of non-aged varietal distillates. 

Some studies prove that the ageing process entails a sensory improvement [37]. However, this does not happen with all varieties in our study. Thus, it is interesting to highlight that the ageing effect differs depending on the grape type, improving the olfactory perception of some of the samples with a lower result as young samples, as happens with the Ugni Blanc and Corredera distillates, which clearly increase their value on the y-axis (Factor 2) after the ageing process (as can be seen, they are located clearly higher in Figure 9), whereas the aged Moscatel de Alejandría and Garrido distillates worsen their global assessment (as can be seen, they change their position in the plane, clearly reducing their coordinates on the y-axis (Factor 2). 

The rest of varietal distillates do not considerably modify their acceptability (related to the value on the y-axis or Factor 2, as commented).

## 4. Conclusions

In this study, 10 distillates from 10 different grape varieties, all of them grown in Jerez, are aged in an accelerated way. The analytical and sensory characteristics of these aged distillates have been determined to identify possible varieties which could represent an alternative to the one currently used for Brandy de Jerez and which does not grow in the area.

The most common polyphenols found in the aged distillates have been gallic acid, syringaldehyde, coniferaldehyde, and sinapaldehyde. On the other hand, color has been evolving in the aged distillates towards a darker tone, corresponding as a result with a lower value of L and H, with a higher yellow-greenish hue. As regards the total polyphenol index, we can observe that, in general, it increases at the beginning to later on decrease and stay stable. The results obtained in these analytical parameters coincide with previous studies.

In the principal component analysis implemented with all studied analytical variables (total polyphenol index, chromatic parameters, and individual polyphenol analysis) on the distillates aged in the accelerated way compared to the Brandy samples aged in the traditional way, a similar behavior in both groups cannot be seen. This could be due to an excessive and direct extraction of a few compounds from the chips in the accelerated ageing method.

However, if we just take into account the principal component analysis carried out with the total polyphenol index and color index comparing the accelerated ageing distillates and the traditionally aged ones, we can see that the distillate made from Airén grapes evolved very quickly, reaching in just one month of accelerated ageing the chromatic and polyphenolic characteristics of a Brandy de Jerez Solera Gran Reserva. Those distillates from the Jaén Blanco, Moscatel de Alejandría, and Zalema grape types behave in a similar way to the traditionally aged Brandy de Jerez Solera Reserva.

The distillates made from these three grape types obtained positive sensorial assessment after being aged, particularly Jaén Blanco and Zalema (both varieties are native from Andalusia and are highly productive), being as a result interesting types of distillates to be taken into account in the future for oenological practice in the area.

As an additional conclusion, we can state that, after checking the analyzed parameters (physical-chemical and sensory), we can infer the essential role played by the used grape varieties in the behavior of the distillate during the ageing stage and, as a result, in obtaining the final product.

## Figures and Tables

**Table 1:** Aromatic descriptors used for the assessment of the samples.

DESCRIPTOR	DEFINITION	Pattern
Aromatic intensity	Intensity of olfactory perception via orthonasal olfaction	Commercial SGR Brandy de Jerez
Fruity	Remainder of raw material (grape)	Young Muscatel distillate
Herbaceous	Sharp green note	Commercial herb liqueur
Vinous	Remainder of recently fermented Sherry wine	Recently fermented Sherry wine (and frozen up to tasting)
Alcoholic	Hot	Varietal Airén distillate of 50°alcohol
Dairy	Characteristic note of the wines which suffer a dairy fermentation	Recent dairy fermentation white wine (and frozen up to tasting)
Sweet	Olfactory perception reminding of caramel	Commercial brandy with added caramel
Oak	Remainder of American oak wood	Brandy aged in the traditional way for 5 years
Vanilla	Sweet note of vanilla pods	Airén distillate with added vanillin
Toasted	Olfactory remainder of tobacco, coffee, cocoa, or smoke	Hydroalcoholic extract of intensely toasted oak chips
Spicy	Remainder of anise, thyme, or clove	Hydroalcoholic extract of a balanced mix of anise, thyme, rosemary, and clove
Chemical character	Olfactory remainder of solvent, glue, or medicine	Hydroalcoholic mix with added aromatic extracts of glue and medicine of Le nez du Vin
Wetness	Earthy olfactory notes, fungus	Hydroalcoholic mix with added fungus extract of Le nez du Vin
Animal	Olfactory remainder of sweat, musk, or leather	Hydroalcoholic mix with added horse sweat, musk, and leather extract of Le nez du Vin
Olfactory impression	General orthonasal perception of the aromatic complexity and intensity and lack of flaws	Commercial SGR Brandy de Jerez

**Table 2:** Results of the two-way analysis of variance applied to IPT and chromatic parameters. Values are expressed as average ± standard deviation according to the variety factor. For each variable, identical superscripts indicate that there is no difference between the values for those varieties. L*: brightness of color and TPI: total polyphenol index.

Variety	TPI (mg L^−1^ Equivalent Gallic Acid)	L*	a (Red-Green)	b (Yellow-Blue)	H (Tone)	C (Chroma)
Airén	(218.28 ± 53.08^) e^	(90.03 ± 3.02^) a^	(−1.12 ± 0.38) ^b^	(31.39 ± 6.38) ^f^	(92.24 ± 1.26) ^a^	(31.41 ± 6.36) ^f^
Colombard	(86.14 ± 10.45) ^c^	(95.1 ± 1.51) ^de^	(−1.7 ± 0.5) ^a^	(24.78 ± 3.61) ^c^	(93.91 ± 1) ^bcd^	(24.85 ± 3.62) ^c^
Corredera	(57.45 ± 3.43) ^ab^	(97.63 ± 0.99) ^f^	(−1.75 ± 0.33) ^a^	(17.04 ± 0.78) ^a^	(95.88 ± 1.05) ^e^	(17.13 ± 0.78) ^a^
Doradilla	(96.65 ± 10.61) ^c^	(94.2 ± 0.77) ^cd^	(−1.64 ± 0.39) ^a^	(30.26 ± 3.26) ^f^	(93.14 ± 0.79) ^b^	(30.31 ± 3.26) ^f^
Garrido	(46.02 ± 3.9) ^a^	(98.08 ± 0.96) ^f^	(−1.9 ± 0.36) ^a^	(18.8 ± 4.24) ^a^	(95.87 ± 0.91) ^e^	(18.9 ± 4.25) ^a^
Jaen Blanco	(146.88 ± 39.82) ^d^	(92.74 ± 1.19) ^b^	(−1.81 ± 0.21) ^a^	(26.09 ± 3.13) ^de^	(94.01 ± 0.46) ^bc^	(26.16 ± 3.13) ^de^
Moscatel	(134.9 ± 18.9) ^d^	(93.73 ± 0.9) ^bc^	(−1.77 ± 0.21^) a^	(23.93 ± 3.59) ^cd^	(94.32 ± 0.83) ^cd^	(23.99 ± 3.59) ^cd^
Palomino Fino	(88.18 ± 9.63) ^c^	(94.31 ± 0.75) ^cd^	(−1.69 ± 0.43) ^a^	(27.47 ± 2.63) ^e^	(93.53 ± 0.93) ^bc^	(27.53 ± 2.62) ^e^
Ugni Blanc	(76.42 ± 10.59) ^bc^	(95.74 ± 0.78) ^e^	(−1.7 ± 0.29) ^a^	(26.65 ± 2.93) ^cde^	(93.69 ± 0.72) ^bc^	(26.71 ± 2.92) ^cde^
Zalema	(148.58 ± 24.34) ^d^	(97.02 ± 0.97) ^f^	(−1.68 ± 0.15) ^a^	(21.1 ± 3.45) ^b^	(94.65 ± 0.74) ^d^	(21.17 ± 3.44) ^b^
*p*_sampling number_	*0.126*	*0.005*	*0.000*	*0.000*	*0.002*	*0.000*
*p*_variety_	*0.000*	*0.000*	*0.000*	*0.000*	*0.000*	*0.000*

**Table 3:** Results of the two-way analysis of variance applied to the individual polyphenols. Values for each variety are expressed as average ± standard deviation (in mg L^−1^). For each variable, identical superscripts indicate that there is no difference between the values for those varieties. (5-HMF: Hydroxymethylfurfuraldehyde; F: Furfuraldehyde; Syringald: Syringaldehyde; Coniferald: Coniferaldehyde; Sinapald: Synapaldehyde).

Variety	Galic Acid	5-HMF	F	Vanillic Acid	Syringic Acid	Vanillin	*p*-Coumaric Acid	Syringald	Coniferald	Sinapald
Airén	(2.55 ± 0.7) ^de^	(0.68 ± 0.19) ^cd^	(1.83 ± 0.39) ^e^	(0.34 ± 0.11) ^c^	(0.79 ± 0.36) ^e^	(0.94 ± 0.26) ^f^	(0.09 ± 0.02) ^ab^	(2.07 ± 0.61) ^c^	(1.72 ± 0.52) ^abc^	(3.86 ± 1) ^ab^
Colombard	(2.54 ± 0.16) ^de^	(0.51 ± 0.04) ^b^	(0.77 ± 0.03) ^b^	(0.2 ± 0.04) ^ab^	(0.42 ± 0.06) ^ab^	(0.79 ± 0.1) ^de^	(0.14 ± 0.02) ^d^	(1.63 ± 0.16) ^b^	(1.98 ± 0.07) ^cd^	(4.63 ± 0.31) ^cd^
Corredera	(2.34 ± 0.4) ^cd^	(0.63 ± 0.05) ^c^	(0.94 ± 0.03) ^c^	(0.21 ± 0.04) ^ab^	(0.51 ± 0.04) ^bcd^	(0.62 ± 0.04) ^ab^	(0.13 ± 0.01) ^d^	(1.35 ± 0.08) ^a^	(1.85 ± 0.15) ^bc^	(4.2 ± 0.53) ^bcd^
Doradilla	(2.74 ± 0.38) ^e^	(0.75 ± 0.04) ^d^	(1.12 ± 0.11) ^d^	(0.34 ± 0.07) ^c^	(0.76 ± 0.09) ^e^	(1.02 ± 0.09) ^f^	(0.13 ± 0.05) ^d^	(1.99 ± 0.25) ^c^	(2.19 ± 0.1) ^d^	(4.62 ± 0.44) ^cd^
Garrido	(2.02 ± 0.2) ^ab^	(0.45 ± 0.09) ^ab^	(0.6 ± 0.1) ^a^	(0.2 ± 0.04) ^ab^	(0.39 ± 0.09) ^ab^	(0.56 ± 0.09) ^a^	(0.1 ± 0.01) ^bc^	(1.28 ± 0.21) ^a^	(1.73 ± 0.14) ^abc^	(3.72 ± 0.53) ^ab^
Jaen Blanco	(2.01 ± 0.2) ^ab^	(0.5 ± 0.1) ^b^	(1.08 ± 0.08) ^d^	(0.25 ± 0.03) ^b^	(0.61 ± 0.08) ^d^	(0.72 ± 0.06) ^cd^	(0.07 ± 0.01) ^a^	(1.87 ± 0.2) ^c^	(1.69 ± 0.44) ^ab^	(4.07 ± 1.05) ^bc^
Moscatel	(2.06 ± 0.23) ^abc^.	(0.41 ± 0.07) ^a^	(0.82 ± 0.07) ^bc^	(0.24 ± 0.03) ^b^	(0.54 ± 0.1) ^cd^	(0.7 ± 0.05) ^cd^	(0.12 ± 0.02) ^cd^	(1.47 ± 0.12) ^ab^	(1.67 ± 0.05) ^ab^	(3.48 ± 0.32) ^a^
Palomino Fino	(2.15 ± 0.13) ^bc.^	(0.62 ± 0.05) ^c^	(0.9 ± 0.03) ^bc^	(0.22 ± 0.04) ^ab^	(0.49 ± 0.06) ^bcd^	(0.65 ± 0.05) ^bc^	(0.12 ± 0.01) ^cd^	(1.46 ± 0.11) ^ab^	(1.75 ± 0.09) ^bc^	(3.97 ± 0.49) ^ab^
Ugni Blanc	(2.22 ± 0.17) ^bc.^	(0.44 ± 0.04) ^ab^	(0.55 ± 0.05) ^a^	(0.23 ± 0.03) ^ab^	(0.46 ± 0.07) ^bc^	(0.83 ± 0.09) ^e^	(0.12 ± 0.01) ^cd^	(1.88 ± 0.17) ^c^	(1.82 ± 0.1) ^bc^	(4.71 ± 0.38) ^d^
Zalema	(1.86 ± 0.13) ^a^	(0.4 ± 0.1) ^a^	(1.09 ± 0.06) ^d^	(0.18 ± 0.04) ^a^	(0.32 ± 0.05) ^a^	(0.59 ± 0.07) ^ab^	(0.12 ± 0.01) ^cd^	(1.47 ± 0.21) ^ab^	(1.49 ± 0.3) ^a^	(3.97 ± 0.86) ^ab^
*p _samplig number_*	0.000	0.173	0.167	0.001	0.006	0.000	0.903	0.000	0.595	0.005
*p _variety_*	0.000	0.000	0.000	0.000	0.000	0.000	0.000	0.000	0.000	0.000
*p _sampling numberxvariety_*	0.954	0.677	0.832	0.829	0.422	0.889	0.511	0.787	0.233	0.366

**Table 4:** Concentrations of polyphenols and furanic compounds of the distillates at the end of the accelerated ageing process.

Compounds	Airén	Colombard	Corredera	Doradilla	Garrido	Jaen Blanco	Moscatel	Palomino Fino	Ungi-Blanc	Zalema
Gallic acid	3.60± 0.05	2.80 ± 0.011	2.30 ± 0.079	3.42 ± 0.135	2.45 ± 0.091	2.05 ± 0.014	2.22 ± 0.091	2.40 ± 0.061	2.33 ± 0.096	2.03 ± 0.059
5-Hydroxymethylfurfuraldehyde	0.86 ± 0.01	0.54 ± 0.004	0.59 ± 0.023	0.76 ± 0.044	0.66 ± 0.015	0.47 ± 0.012	0.44 ± 0.022	0.70 ± 0.015	0.43 ± 0.021	0.34 ± 0.012
Furfuraldehyde	2.34 ± 0.025	0.81 ± 0.003	0.97 ± 0.021	1.10 ± 0.036	0.84 ± 0.039	1.18 ± 0.021	0.88 ± 0.031	0.91 ± 0.036	0.60 ± 0.041	1.09 ± 0.031
Vanillic acid	0.48 ± −0.05	0.23 ± 0.002	0.23 ± 0.005	0.43 ± 0.031	0.30 ± 0.031	0.26 ± 0.006	0.25 ± 0.005	0.29 ± 0.021	0.25 ± 0.034	0.23 ± 0.017
Syringic acid	1.21 ± 0.006	0.54 ± 0.069	0.53 ± 0.004	0.84 ± 0.085	0.61 ± 0.059	0.64 ± 0.004	0.57 ± 0.002	0.61 ± 0.009	0.52 ± 0.017	0.37 ± 0.033
Vanillin	1.27 ± 0.04	0.98 ± −0.010	0.67 ± 0.021	1.15 ± 0.015	0.78 ± 0.053	0.76 ± 0.005	0.72 ± 0.008	0.77 ± 0.030	0.92 ± 0.031	0.69 ± 0.047
*p*-Coumaric acid	0.12 ± 0.03	0.15 ± 0.017	0.13 ± 0.001	0.15 ± 0.008	0.12 ± 0.002	0.08 ± 0.001	0.10 ± 0.036	0.11 ± 0.004	0.13 ± 0.004	0.12 ± 0.022
Syringaldehyde	2.89 ± 0.09	1.84 ± 0.033	1.43 ± 0.031	2.28 ± 0.045	1.77 ± 0.055	1.91 ± 0.018	1.53 ± 0.014	1.71 ± 0.016	2.05 ± 0.011	1.57 ± 0.075
Coniferaldehyde	2.17 ± 0.12	1.93 ± 0.009	1.69 ± 0.051	2.15 ± 0.045	1.91 ± 0.051	1.89 ± 0.001	1.65 ± 0.013	1.69 ± 0.011	1.69 ± 0.033	1.55 ± 0.044
Sinapaldehyde	4.67 ± 0.10	4.23 ± 0.105	3.59 ± 0.061	4.13 ± 0.023	3.50 ± 0.051	4.05 ± 0.031	3.18 ± 0.028	3.47 ± 0.004	4.11 ± 0.027	3.80 ± 0.093

**Table 5:** Principal components analysis. Factor loadings of the first two components extracted. CP: compound.

	CP1: 64.35%	CP2: 19.96%
TPI	0.506557	0.589508
L*	−0.924628	−0.131875
a	0.346001	0.776631
b	0.934685	−0.334195
H*	−0.940139	0.066660
C*	0.933785	−0.336515

**Table 6:** Average scores (±standard deviation) given by the tasting panel for the samples regarding each of the olfactory descriptors used for their sensory assessment.

Variety	Ageing	Aromatic Intensity	Fruity	Herbaceous	Wine Character	Alcoholic	Dairy	Sweet	Oak	Vanilla	Toasted	Spicy	Chemical Character	Wetness	Animal	Olfactory Impression
Airén	Aged	4.2 ± 0.8	2.2 ± 1.4	1.7 ± 1.5	1.9 ± 1.0	3.5 ± 0.9	0.6 ± 0.3	2.1 ± 0.7	2.4 ± 1.1	1.7 ± 1.5	1.5 ± 1.2	1.2 ± 1	0.5 ± 1.0	0.9 ± 0.9	0.6 ± 0.6	3.7 ± 1.3
Colombard	Aged	4.1 ± 0.7	2 ± 1.1	1.0 ± 0.4	1.9 ± 1.0	3.7 ± 0.4	0.4 ± 0.5	2.5 ± 1.0	2.8 ± 0.8	2.6 ± 1.0	1.7 ± 0.8	0.9 ± 0.5	0.7 ± 0.6	0.2 ± 0.4	0.5 ± 0.7	4.6 ± 1.2
Corredera	Aged	4.2 ± 0.6	2.6 ± 1.1	0.9 ± 1.0	1.9 ± 0.7	3.2 ± 0.2	0.5 ± 0.3	2.7 ± 0.5	2.6 ± 0.7	2.5 ± 1.6	1.6 ± 0.8	1.5 ± 1.2	0.5 ± 0.9	0.2 ± 0.4	0.3 ± 0.4	4.8 ± 1.2
Doradilla	Aged	4.4 ± 0.5	2.1 ± 1.5	0.9 ± 0.5	2.0 ± 1.2	3.3 ± 0.8	0.7 ± 0.8	2.6 ± 0.9	3.3 ± 1.0	2.9 ± 1.2	2.2 ± 0.9	1.1 ± 1.1	0.5 ± 0.7	0.3 ± 0.7	0.3 ± 0.3	4.8 ± 1.4
Garrido	Aged	4.1 ± 0.7	2.2 ± 1.4	1.2 ± 0.6	1.7 ± 1.1	3.3 ± 1.0	0.3 ± 0.2	2.4 ± 0.9	2.0 ± 0.7	1.8 ± 1.2	1.2 ± 0.8	1.2 ± 0.9	0.5 ± 0.9	0.4 ± 0.7	0.8 ± 0.3	3.7 ± 0.6
Jaen Blanco	Aged	4.2 ± 0.8	2.3 ± 0.8	1.2 ± 0.2	1.8 ± 1.1	3.5 ± 0.8	0.5 ± 0.1	1.9 ± 1.0	2.4 ± 0.7	1.9 ± 0.9	1.3 ± 1.1	1.4 ± 0.9	0.6 ± 0.6	0.4 ± 0.7	0.5 ± 0.1	4.0 ± 1.1
Moscatel	Aged	5.2 ± 0.9	3.3 ± 1.5	1.2 ± 0.4	2.0 ± 1.0	3.6 ± 0.3	0.4 ± 0.5	2.6 ± 1.4	2.6 ± 0.5	2 ± 1.1.0	1.4 ± 0.7	1.6 ± 1.0	0.9 ± 0.2	0.2 ± 0.4	0.4 ± 0.7	4.6 ± 1.1
Palomino Fino	Aged	4.4 ± 0.4	2.2 ± 1.4	0.9 ± 0.2	1.9 ± 1.1	2.9 ± 1.2	0.7 ± 0.7	2.5 ± 1.3	2.9 ± 1.1	2.4 ± 0.8	1.4 ± 0.7	1.4 ± 1.1	0.5 ± 0.9	0.3 ± 0.4	0.7 ± 0.8	4.8 ± 0.7
Ugni Blanc	Aged	4.0 ± 0.5	2.3 ± 1.0	1.3 ± 0.8	1.9 ± 1.1	3.5 ± 0.2	0.6 ± 1.0	2.4 ± 0.9	2.0 ± 0.6	2.7 ± 1.0	1.4 ± 0.8	1.1 ± 0.7	0.4 ± 0.5	0.7 ± 1.0	0.5 ± 0.7	4.4 ± 0.5
Zalema	Aged	4.4 ± 0.5	2.5 ± 1.1	0.7 ± 0.4	1.6 ± 0.8	3.4 ± 0.3	0.7 ± 0.6	2.5 ± 1.1	2.5 ± 0.5	2.5 ± 0.8	1.3 ± 0.8	1.3 ± 0.8	0.5 ± 0.7	0.6 ± 0.9	0.5 ± 0.9	4.5 ± 1.3
Airén	Young	4.8 ± 1.3	2.8 ± 1.4	0.9 ± 0.8	1.8 ± 0.8	4.2 ± 1.3	0.8 ± 0.6	1.2 ± 1.1	1.0 ± 1.4	1.7 ± 1.6	1.4 ± 1.3	1.2 ± 1.0	1.0 ± 0.5	0.8 ± 0.8	0.0 ± 0.0	4.1 ± 1.2
Colombard	Young	4.6 ± 0.9	3.4 ± 1.5	1.2 ± 0.5	2.0 ± 1.2	3.8 ± 1.1	0.4 ± 0.4	2.6 ± 0.8	1.8 ± 1.9	1.6 ± 1.1	1.4 ± 1.3	1.4 ± 1.3	1.0 ± 0.4	0.2 ± 0.4	0.4 ± 0.9	4.4 ± 1.5
Corredera	Young	4.2 ± 1.2	2.8 ± 1.3	1.4 ± 0.7	2.0 ± 0.8	3.4 ± 0.5	0.4 ± 0.2	2.4 ± 1.4	2.0 ± 2.1	2.2 ± 1.4	0.8 ± 1.1	1.4 ± 1.3	0.8 ± 1.0	0.2 ± 0.4	0.1 ± 0.3	4.4 ± 1.1
Doradilla	Young	4.6 ± 1.3	3.0 ± 0.7	1.4 ± 0.4	1.8 ± 1.1	4.0 ± 0.1	0.1 ± 0.2	2.2 ± 1.1	1.4 ± 1.9	1.6 ± 1.5	1.0 ± 1.4	1.4 ± 0.8	0.8 ± 0.7	0.3 ± 0.0	0.0 ± 0.0	4.4 ± 1.5
Garrido	Young	4.2 ± 1.3	3.5 ± 1.1	1.4 ± 0.4	2.0 ± 1.2	3.4 ± 0.9	0.4 ± 0.6	2.1 ± 1.4	1.4 ± 1.9	1.2 ± 1.6	1.0 ± 1.0	1.6 ± 1.1	1.0 ± 0.7	0.2 ± 0.4	0.0 ± 0.0	4.2 ± 1.2
Jaen Blanco	Young	5.4 ± 1.3	3.4 ± 1.1	1.0 ± 0.8	2.0 ± 1.2	4.0 ± 1.2	0.2 ± 0.4	1.7 ± 1.0	1.2 ± 1.6	1.0 ± 1.4	0.6 ± 0.9	0.8 ± 0.8	0.6 ± 1.0	0.3 ± 0.0	0.1 ± 0.4	4.2 ± 1.5
Moscatel	Young	5.2 ± 0.8	4.6 ± 1.5	1.6 ± 0.9	2.2 ± 1.1	3.7 ± 0.5	0.6 ± 0.5	2.5 ± 1.4	1.8 ± 2.2	1.0 ± 1.4	0.8 ± 1.3	1.0 ± 0.8	0.8 ± 0.5	0.4 ± 0.5	0.2 ± 0.4	5.2 ± 0.8
Palomino Fino	Young	4.4 ± 0.5	3.4 ± 0.5	1.2 ± 0.6	2.2 ± 1.1	3.6 ± 1.1	0.2 ± 0.4	2.2 ± 1.0	1.4 ± 1.9	1.4 ± 1.5	1.0 ± 1.4	1.0 ± 0.7	0.4 ± 0.9	0.2 ± 0.4	0.2 ± 0.4	4.4 ± 1.1
Ugni Blanc	Young	3.8 ± 0.4	2.6 ± 0.5	0.8 ± 0.2	2.0 ± 0.8	3.6 ± 1.1	0.2 ± 0.4	1.8 ± 1.0	0.4 ± 0.9	0.4 ± 0.9	0.8 ± 1.1	1.0 ± 1.1	1.2 ± 1.0	0.3 ± 0.3	0.6 ± 0.9	3.0 ± 0.2
Zalema	Young	4.2 ± 1.3	3.4 ± 1.2	1.4 ± 0.3	2.0 ± 0.7	3.5 ± 0.4	0.4 ± 0.7	2.1 ± 0.7	1.4 ± 2.2	1.8 ± 1.0	0.8 ± 1.1	1.0 ± 0.4	0.8 ± 1.1	0.5 ± 0.7	0.0 ± 0.0	4.4 ± 0.8
ANOVA Assessors × Samples																
*p*_Assessors_		0.723	0.558	0.091	0.644	0.125	0.414	0.109	0.102	0.279	0.118	0.229	0.277	0.459	0.212	0.605
*p*_Assessors × Samples_		0.334	0.230	0.489	0.762	0.764	0.838	0.112	0.239	0.271	0.498	0.669	0.809	0.236	0.311	0.552
ANOVA Varieties × Ageing																
*p*_Varieties_		0.491	0.078	0.813	0.867	0.429	0.662	0.651	0.779	0.656	0.882	0.287	0.698	0.226	0.737	0.445
*P*_Ageing_		0.211	*0.026*	*0.028*	0.102	*0.031*	0.205	*0.044*	*0.003*	*0.033*	*0.021*	0.457	*0.037*	0.128	*0.033*	0.766
*p*_Varieties × Ageing_		0.187	0.744	0.047	0.334	0.232	0.188	0.072	0.879	0.085	0.101	0.222	0.066	0.102	0.069	0.326
